# Imaging of Strictures in Crohn’s Disease

**DOI:** 10.3390/life13122283

**Published:** 2023-11-29

**Authors:** Laura Maria Minordi, Luigi Larosa, Antonio Bevere, Francesca Bice D’Angelo, Antonio Pierro, Savino Cilla, Annemilia Del Ciello, Franco Scaldaferri, Brunella Barbaro

**Affiliations:** 1 Dipartimento di Diagnostica per Immagini, Radioterapia Oncologica ed Ematologia, Fondazione Policlinico Universitario A. Gemelli IRCCS, Largo A. Gemelli 8, 00168 Roma, Italy; lauramaria.minordi@policlinicogemelli.it (L.M.M.); annemilia.delciello@policlinicogemelli.it (A.D.C.); brunella.barbaro@policlinicogemelli.it (B.B.); 2 Università Cattolica del Sacro Cuore, L.go F. Vito 1, 00168 Rome, Italy; antonio.bevere@hotmail.com (A.B.);; 3 Radiology Unit, San Timoteo Hospital, 86039 Termoli, Italy; antonio.pierro@asrem.org; 4 Medical Physics Unit, Responsible Research Hospital, 86100 Campobasso, Italy; savinocilla@gmail.com; 5 CEMAD Digestive Diseases Center, Fondazione Policlinico Universitario A. Gemelli IRCCS, Largo A. Gemelli 8, 00168 Rome, Italy; franco.scaldaferri@policlinicogemelli.it

**Keywords:** bowel stricture, Crohn’s disease, CT enterography, MR enterography

## Abstract

Crohn’s disease (CD) is a chronic inflammation of the digestive tract, and it frequently affects young patients. It can involve any intestinal segment, even though it frequently affects the distal ileum. Up to 80% of patients with CD present with inflammatory behavior, and 5% to 28% develop stricturing disease. Based on the predominant mechanism causing them, strictures can be categorized as inflammatory, fibrotic, or mixed. Determining the relative amounts of inflammation and fibrosis in a stricture can influence treatment decisions. Imaging is an extremely useful tool in patients with small bowel stricturing CD to confirm the diagnosis and to evaluate disease characteristics, usually using CT or MRI. The aim of this paper is to describe how imaging can evaluate a patient with small bowel CD stricture.

## 1. Introduction

Crohn’s disease (CD) is a chronic inflammation of the digestive tract, and it frequently affects young patients (second and third decade). It can involve any intestinal segment, from mouth to anus, even though it frequently affects the distal ileum [1]. It is usually suspected on the basis of clinical and laboratory findings, as it causes abdominal pain, weight loss, diarrhea, and fever, and it is accompanied by an increase in blood inflammation markers such as C-reactive protein. The diagnosis must subsequently be confirmed using endoscopy with biopsy [2].

Up to 80% of patients with CD present with inflammatory behavior, and 5% to 28% develop stricturing disease [3]. Stricturing CD usually results from intestinal fibrosis; however, inflammation is necessary for establishing fibrosis, but it plays a minor role in its progression. Based on the predominant mechanism causing them, strictures can be categorized as inflammatory, fibrotic, or mixed. Determining the relative amounts of inflammation and fibrosis in a stricture can influence treatment decisions. In fact, inflammatory strictures are initially managed with medical therapy (corticosteroids, anti-Tumor Necrosis Factor-TNF, or other monoclonal antibodies and immunosuppressants). Fibrotic strictures, on the other hand, are generally managed operatively, either endoscopically (endoscopic balloon dilatation or stenting) or surgically (stricture-plastic or intestinal resection). At present, there are no approved or effective medical therapies against intestinal fibrosis [4].

Imaging is an extremely useful tool in patients with small bowel stricturing CD to confirm the diagnosis and to evaluate disease characteristics, usually using Computed Tomography (CT) or Magnetic Resonance Imaging (MRI). CT has high spatial resolution, but on the other hand it gives ionizing radiations. For this reason, MRI is preferred in the follow-up of young patients. CD manifests itself in the form of intestinal wall thickening, of variable degree, which causes a reduction in the caliber of the lumen with or without dilatation of the upstream loops. Wall thickening can be of various degrees (mild, moderate, and marked), and dilatation upstream of a stenosis consists of three degrees, with a dilated loops caliber greater than 4 cm in the most severe forms.

The aim of this paper is to describe how imaging can evaluate a patient with small bowel CD stricture, answering the following clinical questions: (1) What is the best technique to request for the evaluation of a patient with small bowel stricture? (2) Is there an intestinal stricture? (3) What is the site of the stricture? (4) How extensive is the disease? (5) Is the stricture fibrotic or inflammatory? (6) Is there another cause of stricture? (7) What evaluation can be performed after therapy?

## 2. What Is the Best Technique to Request for the Evaluation of a Patient with Small Bowel Stricture?

**Figure 1:**
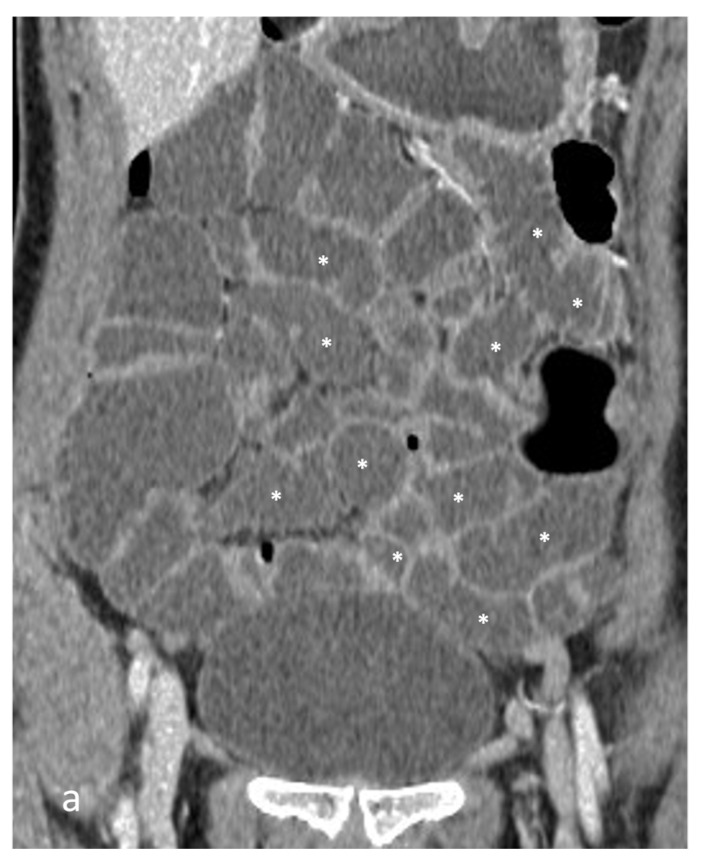

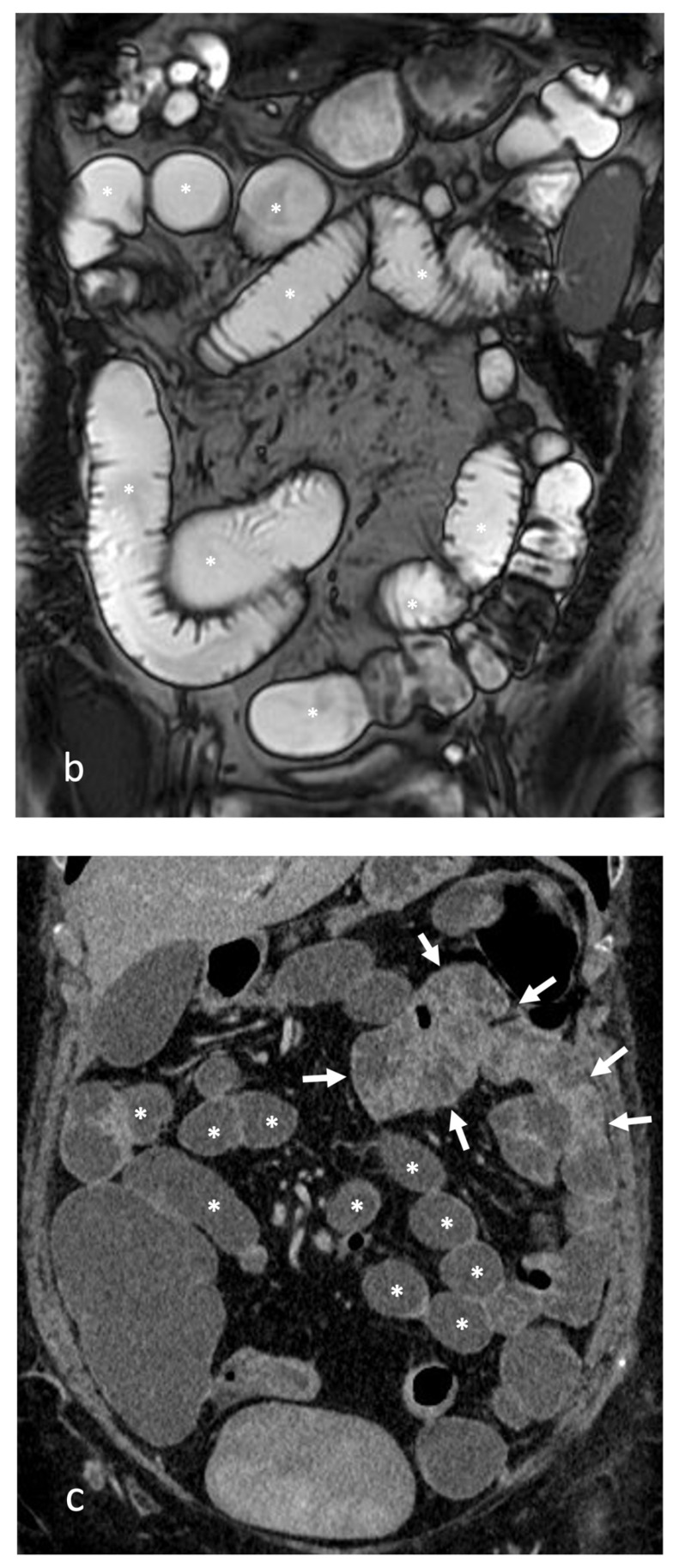
Small bowel distension in CT and MR enterography after oral administration of polyethylene glycol solution. (**a**) Coronal CT and (**b**) coronal T2-weighted MR images show great distention of the small bowel loops (asterisks), including both ileum and jejunum. (**c**) Coronal CT image of another patient shows good distention of the ileal loops (asterisks), whereas the jejunum is collapsed in the left hypochondrium (arrows).

Patients with CD can be studied with different imaging techniques, including conventional radiological examinations, ultrasound, and FDG PET-CT, but CT or MRI are the most validated ones for stricture evaluation. In both cases, it is necessary to obtain distension of the small bowel loops with a contrast medium agent (polyethylene glycol solution, oil emulsions, water, air, Mucofalk, dilute barium sulfate, mannitol, sorbitol, or locust bean gum) that can be administered orally in MR or CT enterography (CTE, MRE) or through a naso-jejunal tube in MR or CT enteroclysis. These agents highlight the CT/MR density/intensity differences between the bowel lumen and the bowel wall, helping to spot its pathological thickenings and ulcerations [5,6,7]. However, oral administration of contrast agents causes variable distension of small bowel loops; this variability depends on patient compliance, the amount of contrast agent drunk, the time taken, and any previous surgery. The ileum is typically better distended than the jejunum, especially in patients who still have the ileocecal valve (Figure 1) [5,6,7,8,9]. PEG is one of the oral contrast mediums most commonly used for small bowel distension, due to its low cost and few side effects, and it is usually administered in doses of 100 mL starting 35–50 min before the MR or CT examination, reaching a total volume of 1–2 L.

CT examination generally consists of a single image acquisition after bowel distension and 75–80 s after intravenous injection of an iodinated contrast agent.

Conventional MRE sequences usually performed in a patient with CD are described in Table 1. Single-shot T2-weighted images and balanced steady-state free precession (bSSFP) sequences, both in axial and coronal planes, are effective in visualizing the bowel wall, mesentery, and extra-intestinal structures. Axial T2-weighted fat-suppressed images can detect bowel wall edema and intra-abdominal fluid collections, both with a hyperintense signal compared to an adjacent muscle (for example the psoas muscle). Cinematic thick slab coronal bSSFP images assess peristalsis and help to differentiate between under-distended and inflamed bowel loops. Coronal multiphase 3D T1-weighted fat-suppressed post-contrast images, taken 45 and 75 s after an intravenous contrast agent injection, can allow study the intestinal mural enhancement pattern and mesenteric vascularity, identifying the inflamed loops. Delayed axial T1-weighted fat-suppressed images, taken 120 s after an intravenous contrast agent injection, can help detect complications such as fistulae and abscesses. The newer diffusion-weighted imaging (DWI) sequence, performed with multiple b values (usually 0–800 s/mm^2^ or 0–600 s/mm^2^), in coronal or axial planes, is used to support the detection of bowel wall inflammation and extra-luminal fluid collection.

The administration of a contrast medium is also necessary for evaluating the characteristics of mural contrast enhancement; it is used as an iodinate contrast medium for CT examinations and a paramagnetic contrast medium for MRI.

When possible, it is useful to inject an intravenous anticholinergic agent to inhibit peristalsis and reduce related motion artifacts of the bowel, avoid spasms, reach homogeneous distension of the small intestine, and decrease a patient’s abdominal discomfort. Usually 20 mg of N-butyl-hyoscine bromide or 1 mg of Glucagon are injected before the contrast sequences, especially during MR examinations, which notoriously last longer and are therefore more subject to this type of artifact.

**Figure 2:**
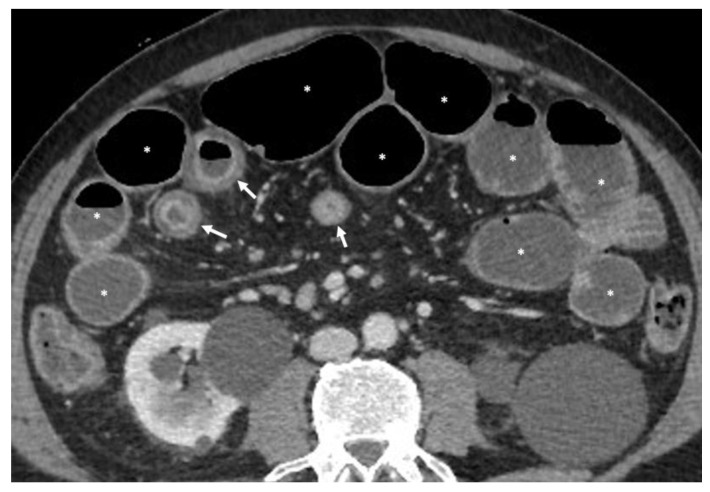
Axial CT image after injection of contrast medium, performed at the emergency department, shows wall thickening of some small bowel loops in right abdomen (arrows), due to Crohn’s disease, which is causing upstream bowel dilatation (asterisks).

In patients presenting to the emergency department with acute manifestations of intestinal obstruction, an abdominal CT with a contrast medium and without distension of the loops should be performed and is useful for evaluating the disease (Figure 2) [10].

In the literature, some studies have evaluated the accuracy of CTE and MRE for the diagnosis of the stricture affecting the small bowel [2].

The sensitivity and specificity ranges of MRE for the identification of strictures are similar to those of CTE, with 75–100% and 91–96%, respectively, depending on the reference standard reported in the papers [11].

In studies comparing CTE to ileocolonoscopy as the reference standard, CTE showed a sensitivity of 92% and a specificity of 100% for the detection of strictures [2,12,13,14]. Other studies, which used endoscopy and surgery as reference standards, reported CTE sensitivities of 85% and 90%, respectively, with a specificity of 100% [2,15,16].

MRE studies with endoscopy and/or surgery such as the reference standard demonstrated a sensitivity of 89% and a specificity of 94% [2,17].

The MR diagnostic accuracy improved when a contrast medium agent was introduced through a naso-jejunal tube rather than orally, with sensitivity rates of 100% and 86% and specificity rates of 100% and 93% for enteroclysis and enterography, respectively [18].

## 3. Is There a Small Bowel Stricture?

The exact definition of a stricture has not been precisely established, varying across different studies; it usually involves luminal stricture with intestinal wall thickening without pre-stenotic dilatation, luminal stenosis with intestinal wall thickening and with pre-stenotic dilatation, and lesion-causing residual lumen < 1 cm [19].

However, when MRE or CTE must be evaluated, the following key aspects of pathological small bowel tracts should be defined: (a) grade of small bowel wall thickening, (b) length of the affected bowel tract, (c) minimum caliber of the lumen, and (d) pre-stenotic bowel dilatation.

**Figure 3:**
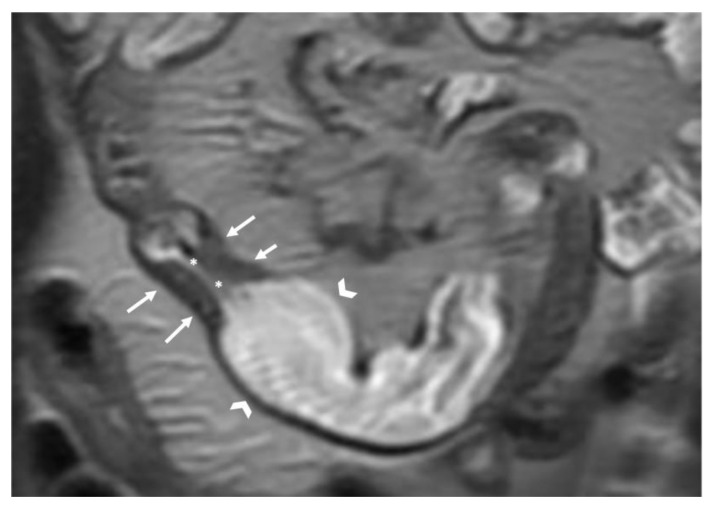
Coronal T2-weighted image shows bowel wall thickening (arrows), determining lumen stenosis (asterisks) and marked upstream dilatation (arrowheads).

1. The wall of a small bowel loop is defined thickened when its thickness (measured from mucosal to serosal layers) is more than 3 mm. According to the degree of thickness, small bowel thickening is defined as “mild” if the thickness is less than 1 cm, “moderate” if it is between 1 cm and 2 cm, and “marked” if it is more than 2 cm [20,21].
2. Regarding the length of the bowel tract affected, a stricture is considered focal if it is less than 5 cm long, segmental if it is between 6 and 40 cm, and diffuse if it is longer than 40 cm [20,21,22]. In the case of multiple stenotic tracts, the healthy tracts interposed between the stenotic ones must not be considered.
3. The minimum caliber of the lumen of the bowel loop affected by the stricture is typically considered pathological if it is less than 10 mm at the site of bowel wall thickening or if it is less than 50% compared to the adjacent bowel tracts [23]. Obviously, a correct valuation of this caliber presupposes good bowel preparation, in order to exclude false positives due to inadequate bowel loop distension.
4. A normal bowel loop caliber ranges between 2 and 2.5 cm. A bowel lumen is dilated when it has a maximum diameter greater than 2.5–3 cm. The dilation is mild when the upstream lumen is dilated up to 4 cm and severe when it is more than 4 cm (Figure 3) [24]. Pre-stenotic bowel dilatation should always be checked, as it is a sign related to bowel obstruction. Moderate to severe stenosis was determined via double-contrast imaging (conventional barium study) with a sufficient amount of injected air, and stenosis was defined as stenosis in which the lumen was less than one half that of neighboring healthy intestine [25].

## 4. Where Is the Stricture?

The site of stricture follows the same distribution of inflammation; therefore, it can involve any intestinal segment, even though it more frequently affects the distal ileum [1]. Approximately 40–55% occur in the terminal ileum and colon, 15–25% in the colon only, 25–40% exclusively in the ileum, and up to 10% in the upper gastrointestinal tract [1]. We defined the anatomy of the small bowel loops in the coronal reformat images. The small bowel loops occupy the infra-mesocolic space of the peritoneal cavity. The jejunal loops are usually arranged in the left upper and mid-quadrants while the ileal loops are in the right mid- and lower quadrants of the abdominal cavity. The proximal jejunum is folded in the left upper quadrant, positioning almost vertically. The distal jejunum crosses the midline from the left to the right side and continues with the proximal ileal loops. Most of the ileal loops are arranged in the space above the pelvic inlet. The distal ileal loops are usually orientated upwards and to the right. The jejunum is usually more anterior than the ileum in the abdominal cavity.

CT/MRI can identify the exact site of the small bowel disease (terminal/distal/proximal ileum, distal, and proximal jejunum) and, when possible, they can measure the distance from the ileo-cecal valve or Treitz ligament. Describing the distance of the stricture from the ileocecal valve helps with surgical planning, potentially modifying the surgical approach and determining the extent of bowel resection needed. This information is especially important for those patients who have undergone previous surgery, for which they risk short bowel syndrome. Furthermore, strictures located close to the ileocecal valve may be more suitable for stricturoplasty, whereas those located further along the bowel may require more extensive resection [26].

## 5. How Extensive Is the Disease?

Currently, there is no manual, semi-automatic or automatic software dedicated to the evaluation of small bowel loops. The curved anatomy and its irregular distribution and distension in the abdominal cavity make the design of dedicated software complicated.

**Figure 4:**
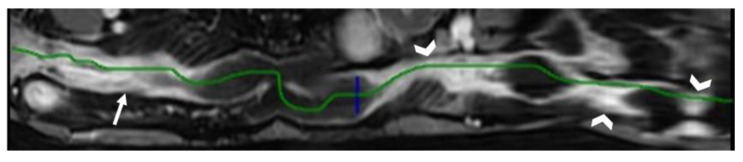
Patient with Crohn’s disease in medical treatment with clinical worsening. MR reconstructed image obtained with dedicated software shows four tracts of distal ileum, characterized by wall thickening and reduced lumen caliber, with stratified contrast enhancement after gadolinium administration, involving both the terminal ileum (arrow) and three more proximal ileal tracts (arrowheads). This reconstruction reveals that these pathological tracts are located in the last 44 cm of ileum, up to the ileocecal valve. The length of each segment is as follows: segment 1 is 11 cm, segment 2 is 8 cm, segment 3 is 3.5 cm, and segment 4 is 3 cm. Luminal narrowing and mild dilatation of the loops between the strictures (maximum caliber of 28 mm) is also observed. Multidisciplinary decision: change of medical therapy.

In our institution, we utilize a vessel analysis software to measure small bowel length in CT enterography or in MR enterography [27]. This method takes advantage of 2D and 3D curved multiplanar reconstructions and allows the stretching of each loop through manual point-by-point identification of intestinal lumen. The creation of a virtual image allows us to quantify the length of the pathological bowel with a linear measurement. In case of multiple bowel wall thickenings, this method also permits reporting of the total length of the bowel affected, from the most proximal to the most distal bowel wall thickening and the length of bowel tracts free from disease between each pathological bowel tract (Figure 4). The time required to carry out the measurements is variable, according to our calculations, from 10 to 20 min for each exam, which adds to the time necessary for viewing the images and writing the report. For this reason, we usually do the aforementioned measurement only for those patients that require surgical planning.

Indications for surgical resection or modification/confirmation of medical therapy are discussed in a multidisciplinary team meeting (MDT), which takes place in our department once a week and includes radiologists, gastroenterologists, general surgeons, and pathologists (Figure 4).

## 6. Is the Stricture Fibrotic or Inflammatory?

CD inflammation and fibrosis are strictly connected mechanisms, which usually coexist in the same patient and even in the same intestinal tract in varying degrees, making the diagnosis even more complex. Therefore, in clinical practice, strictures displaying both fibrotic and inflammatory characteristics are often found. This can be attributed to the presence of a combination of these two components [1].

Imaging, mainly MRI, can help to differentiate predominantly inflammatory from predominantly fibrotic strictures.

**Figure 5:**
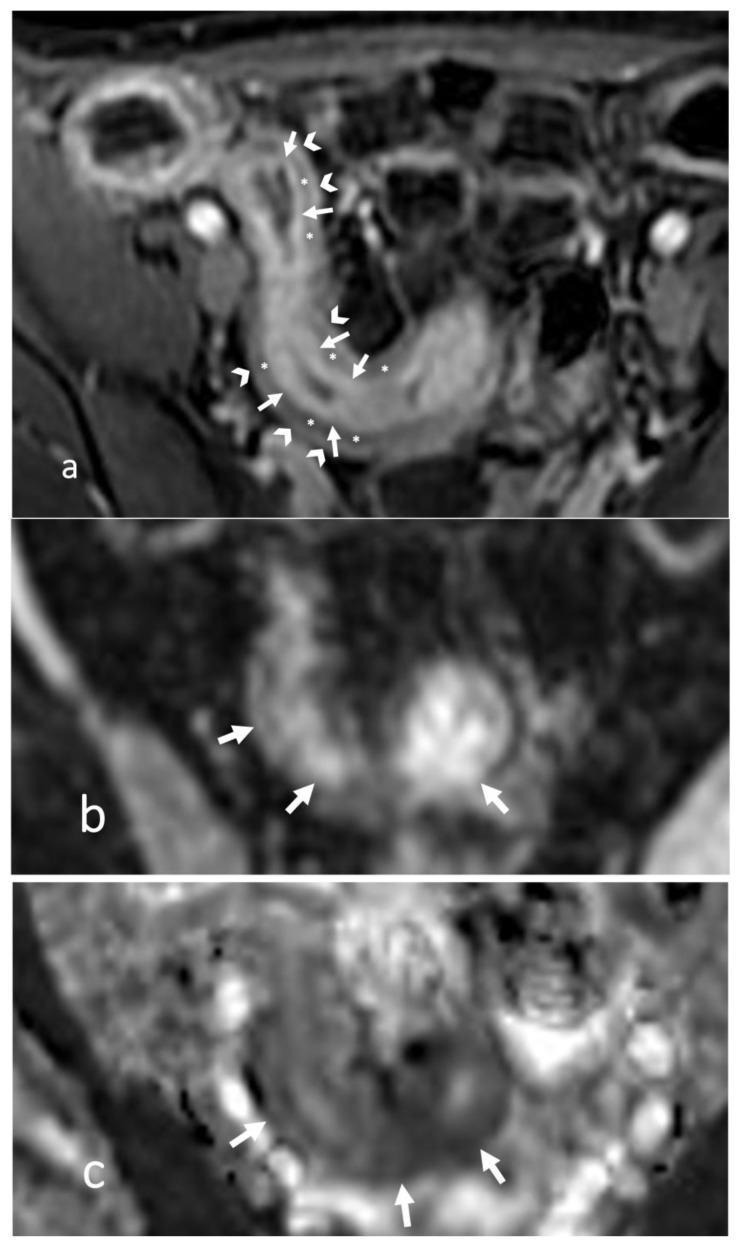

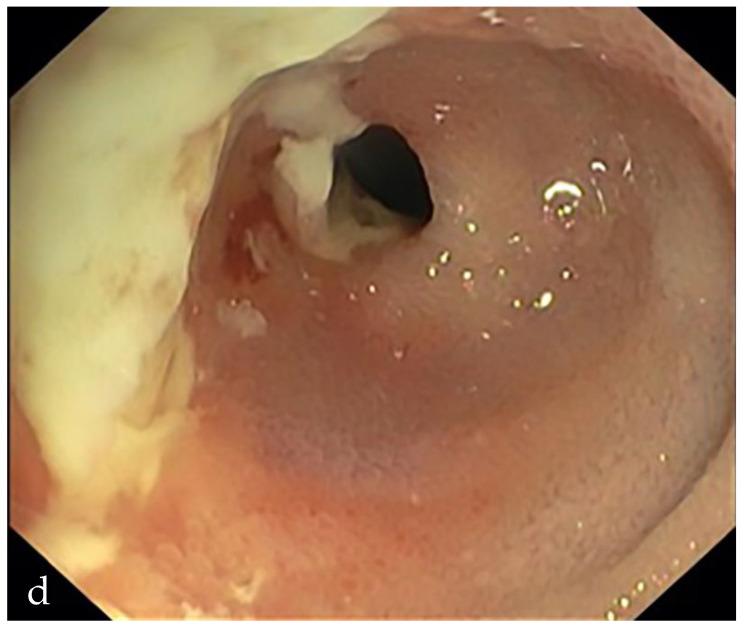
MR appearance of inflammatory strictures. (**a**) Axial T1-weighted image after gadolinium injection demonstrates the typical stratified contrast enhancement pattern of an inflamed bowel loop. It is characterized by hyperenhancement of mucosa (arrows) and serosa (arrowheads) and by hypo-intensity of the submucosal layer (asterisks). (**b**,**c**) DWI image and ADC map of the same bowel loop show restricted diffusion (arrows), confirming the hypothesis of active inflammation. (**d**) Endoscopic appearance of the inflammatory stricture in Crohn’s disease of the terminal ileum. The lumen of the ileum is reduced. The mucosa appears ulcerated in up to 50% of the stenotic area. A deep ulcer is departing from that stricture upfront, involving up to 25% of the entire ileal mucosa.

**Figure 6:**
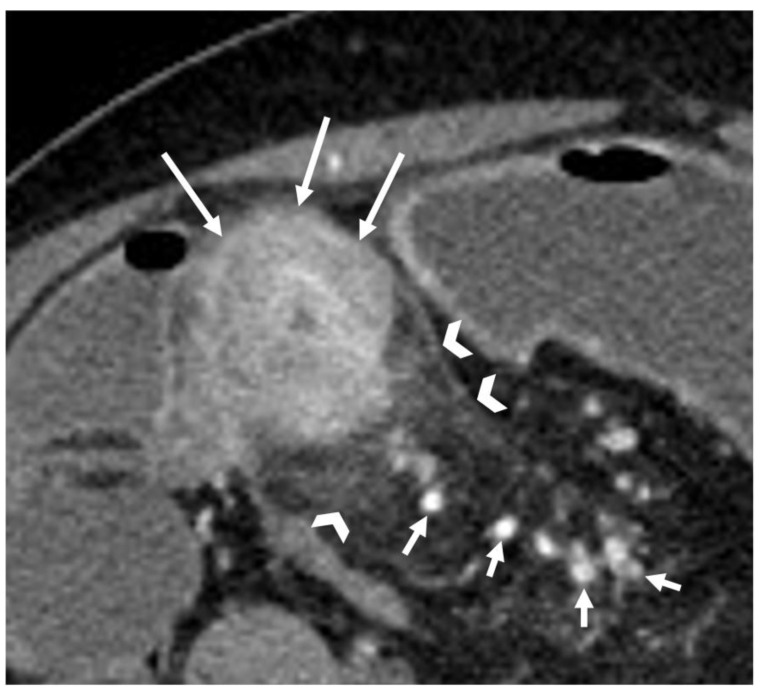
CT appearance of inflammatory strictures. Axial CT image after contrast medium injection shows marked wall thickening of a bowel loop (long arrows), with enlarged vessels (short arrows) in the adjacent mesentery, diffusely hyperdense (arrowheads). These CT features are related to active inflammation.

Imaging features of inflammatory strictures are (Figure 5 and Figure 6): mucosal edema, ulceration, stratified contrast enhancement, DWI restriction (in MRI), and loco-regional hypervascularization.

Mucosal edema can be demonstrated in MRE-T2 fat-saturated images, where the inflamed bowel wall appears slightly hyperintense compared to skeletal muscle. Fat saturation is fundamental to spotting this subtle finding, since in regular T2-weighted sequences the bowel wall hyperintensity may be related to fat infiltration, which is typically seen in CD with chronic, long-standing inflammation (“fat halo sign”) [28].

The presence of ulcerations at the site of the stenosis is another sign of active inflammation, and they are usually seen as an irregularity of the mucosal line, showing focal depressions, sometimes even deep ones.

A layered pattern of contrast enhancement can be seen in both CT and MRI images after contrast medium intravenous injection; it is shown via the hyperenhancement of the inner mucosa and the outer muscle and serosa layer, with an intermediate density/intensity edematous submucosa in between [29].

DWI is a newer sequence that is capable of detecting active inflammation with high accuracy as it is sensitive to changes in tissue cellularity and water diffusion; therefore, an inflamed bowel loop will appear hyperintense in DWI (with respective low ADC values) [30].

Another sign of active inflammation seen after contrast medium injection is locoregional hypervascularization, due to increased blood flow and perfusion in the affected area with a consequent enlarged appearance of vessels in the adjacent mesentery [31].

**Figure 7:**
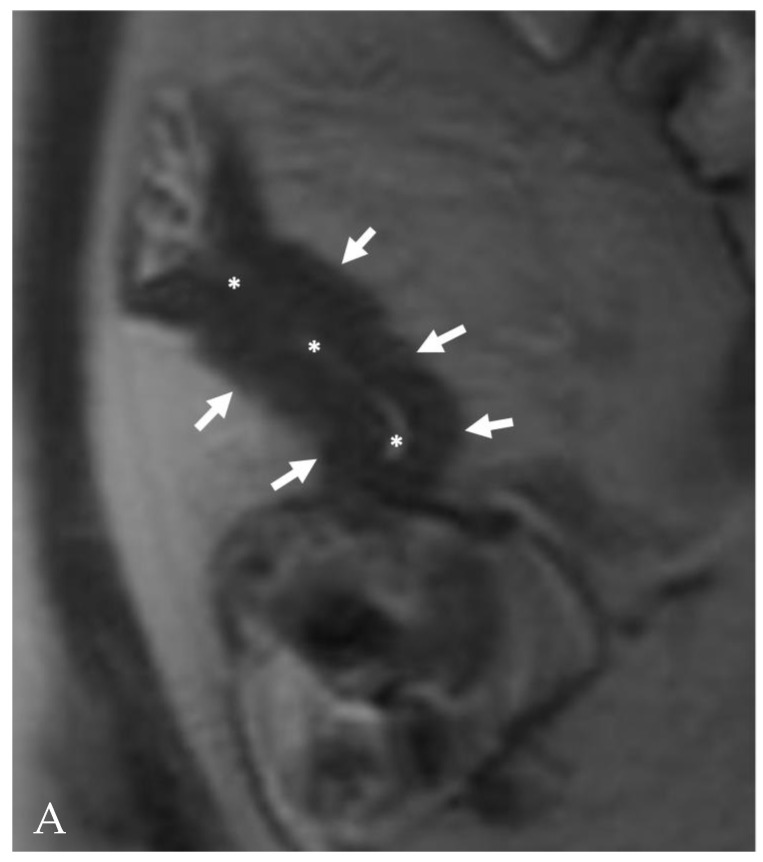

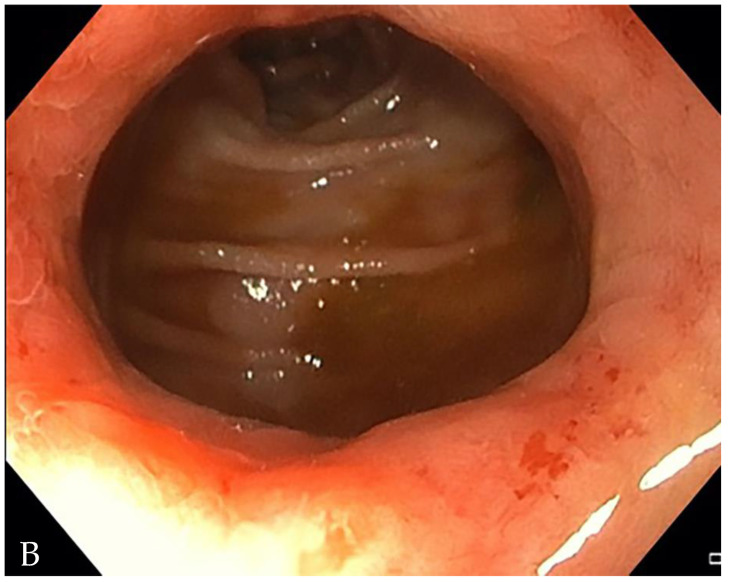
MR appearance of fibrotic strictures. (**A**) Coronal T2-weighted image shows bowel wall thickening, with lumen stenosis (asterisks) and hypointense signal (arrows), lower compared to skeletal muscle signal (not shown in the figure). (**B**) Endoscopic appearance of a fibrotic stricture in Crohn’s disease of the terminal ileum. The lumen of the ileum is reduced: endoscopic exploration of the terminal ileum is not possible. The mucosa appears edematous in absence of ulcers or other signs of active inflammation.

**Figure 8:**
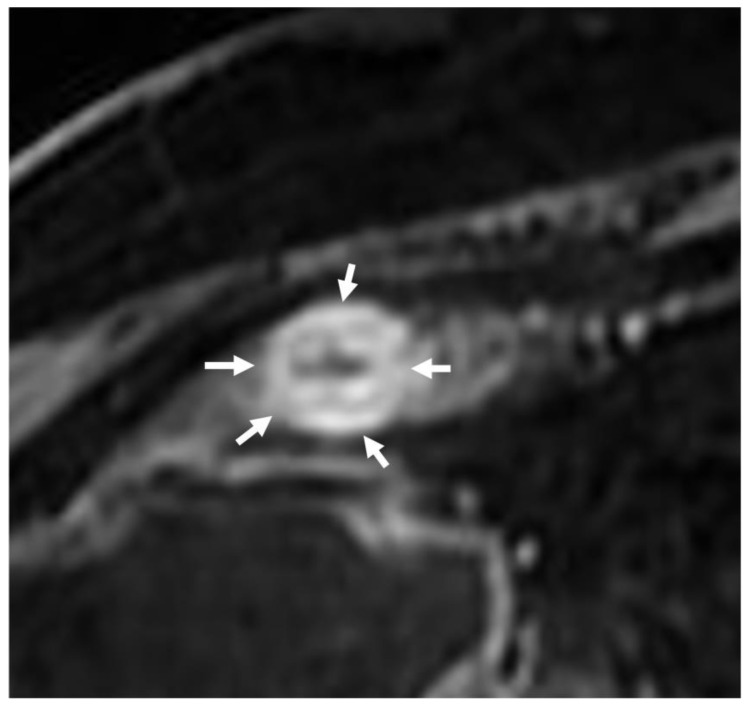
Contrast enhancement MR appearance of fibrotic strictures. Axial T1 image after contrast medium injection shows bowel wall thickening with increased, homogeneous contrast enhancement, usually more prominent in the delayed phase (arrows). This contrast enhancement pattern is related to the presence of fibrosis.

On the other hand, imaging features of fibrotic strictures demonstrate a T2 hypointense intestinal wall signal (Figure 7) and homogenous enhancement after injection of a contrast medium (Figure 8). These imaging features of lower T2 intensity compared to skeletal muscle (instead T2 hyperintensity), and homogenous (instead of layered) contrast enhancement, together with the absence of restricted diffusion, help to distinguish fibrotic stenosis from inflammatory stenosis [29]. In DWI sequences, fibrotic strictures often show low signal intensity [30].

Diffusion kurtosis imaging (DKI) has demonstrated a greater capacity to understand the complex bowel structure in CD patients compared to DWI. Du et al. Researchers [32] evaluated the ability of conventional MRI (T2-weighted parameters) in combination with the DKI parameter Kapp to identify bowel fibrosis in CD patients. They assessed the combination of Kapp and T2 data that could noninvasively distinguish fibrotic strictures from inflammatory ones. However, since fibrosis ranged from moderate to severe in most of the included patients, this study needs to be further checked in patients with early fibrosis.

In CD patients, inflammatory strictures can also be distinguished from fibrotic ones using other tools such as MT ratio of magnetization transfer imaging in association with conventional MRI (T2-weighted sequences) [33] or dynamic contrast-enhanced MRI with intravoxel in coherent motion [34].

In addition to diagnosing strictures, various studies have tried to differentiate between inflammation, fibrosis, and muscularization within a given stricture. Each imaging modality, with single or combined parameters, has been explored, with MRE showing the most promising results in accurately distinguishing between different histopathological stricture components [11,35]. In a study by Fornasa et al., MRE accurately differentiated between active inflammatory strictures (defined by high T2-weighted signal intensity and post-gadolinium T1-weighted enhancement) and fibrotic strictures, allowing for a short-term response to anti-inflammatory therapy in 96% of patients with active inflammatory disease [36].

## 7. Is There Another Cause of Stricture?

CD inflammatory bowel thickening can sometimes present with bowel obstruction causing bowel dilatation before the site of the disease. When assessing strictures, the transition point should be carefully evaluated to establish the cause of the bowel obstruction and exclude other potential differential diagnoses that may present with similar symptoms and findings, such as adhesive disease and bowel cancer [37].

**Figure 9:**
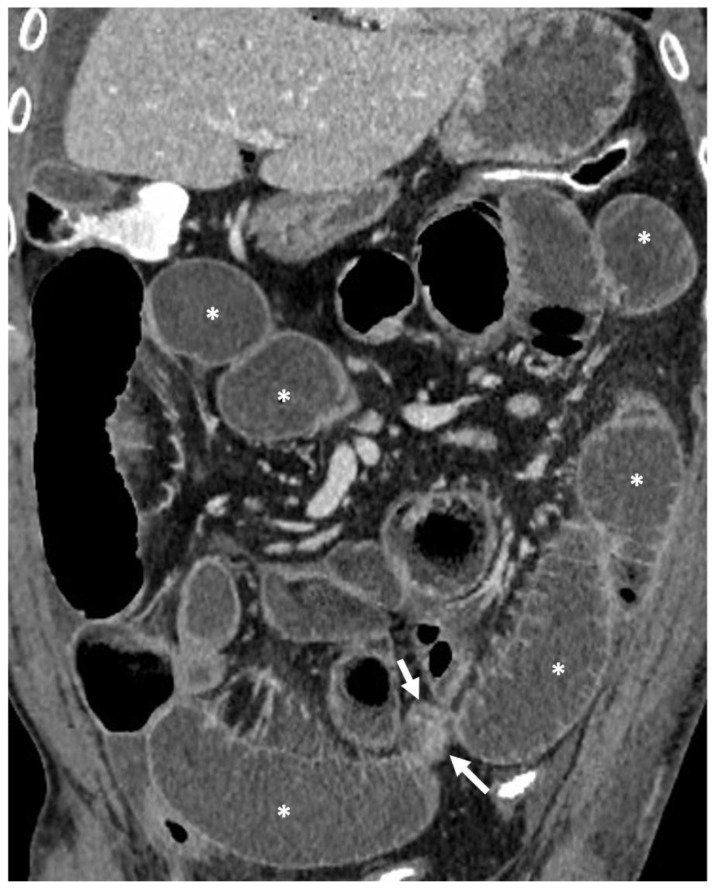
CT appearance of bowel obstruction caused by adhesive disease. Coronal CT image after contrast medium injection, performed at the emergency department, shows bowel dilatation (asterisks) upstream from the transition point (arrows). At the transition point, bowel loops appear angulated and distorted, suggesting the presence of adhesions (surgical confirmation).

**Figure 10:**
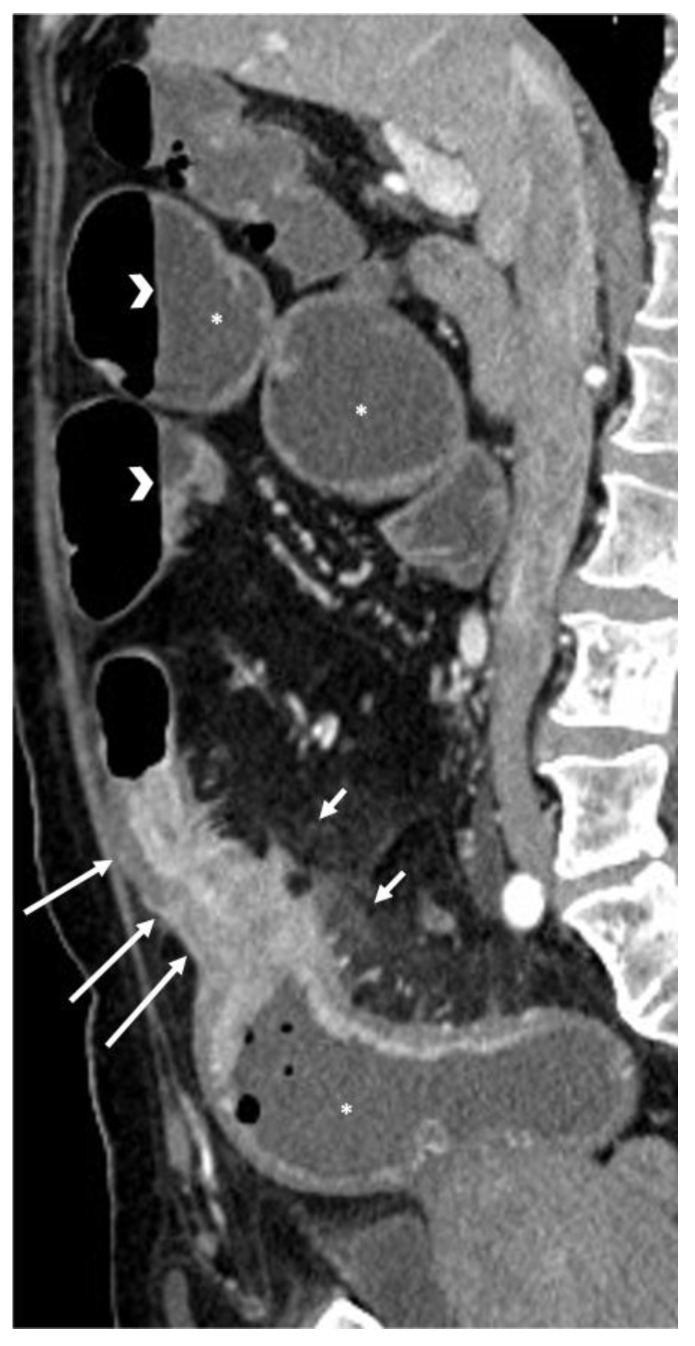
CT appearance of bowel obstruction caused by Crohn’s disease. Sagittal CT image after contrast medium injection, performed at the emergency department, shows Crohn’s disease-related bowel wall thickening (long arrows), causing upstream bowel dilatation (asterisks) with associated air–fluid levels (arrowheads). Hyperdensity of the mesentery adjacent to the bowel wall thickening is also evident (short arrows).

Adhesive disease (Figure 9), as well as Crohn’s disease (Figure 10), can cause bowel obstruction and show bowel dilatation before the transition point site of the adhesions. Imaging cannot directly visualize abdominal adhesions. In fact, its diagnosis is indirect, and it consists in identifying collapsed and distorted bowel segments at the transition point, without evidence of parietal or extra-visceral alterations. Clinical history of prior multiple abdominal surgery or radiation therapy may help in the differential diagnosis.

**Figure 11:**
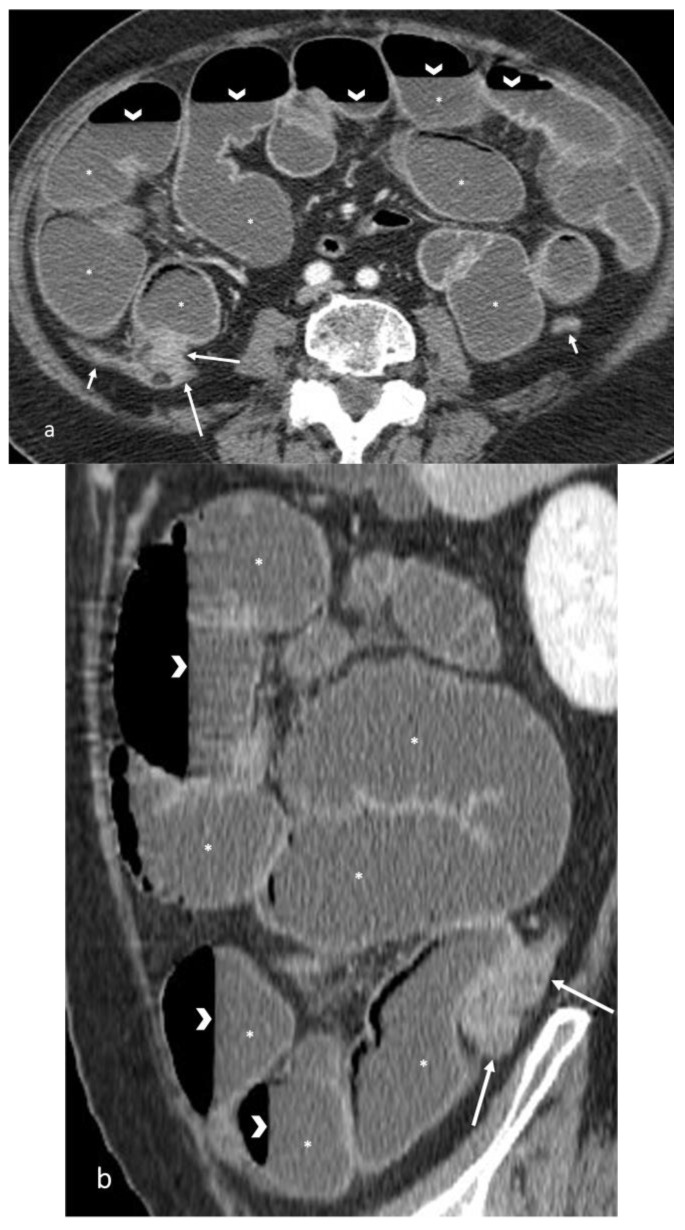
CT appearance of bowel obstruction caused by bowel cancer. (**a**) Axial and (**b**) sagittal CT images after contrast medium injection, performed at the emergency department, show irregular bowel wall thickening (long arrows), involving the bowel at the ileocecal valve, related to bowel cancer, causing marked dilatation of upstream bowel loops (asterisks) with multiple air–fluid levels (arrowheads). The downstream colon loops are collapsed (short arrows).

Bowel cancer may present as a bowel wall thickening and must be differentiated from CD (Figure 11). In the case of malignant bowel disease, wall thickening is more likely focal, asymmetrical, and marked, with inhomogeneous enhancement after contrast medium injection. However, a marked thickening can be also observed in benign conditions, such as severe inflammation or serious infections.

Therefore, clinical features and laboratory findings are fundamental for a correct differential diagnosis.

## 8. What Evaluation to Use after Treatment?

It has been hypothesized that in patients with strictures a treatment with steroids or other treatments, as monoclonal antibodies, could reduce the inflammatory process within the stricture. However, some studies showed that use of steroids was significantly associated with stricture appearance and a higher rate of complications after surgery [38].

At present, an antifibrotic drug for bowel strictures does not exist. In recent decades, the use of azathioprine did not reduce the incidence of stricture appearance and the surgical rate in patients with CD, with the number of surgical interventions performed per year ranging between 3.3% and 7.5% three months after diagnosis. Azathioprine was demonstrated to act effectively on the inflammatory process at the anastomosis and to maintain remission in up to 70–80% of patients with CD. However, azathioprine/6-mercaptopurine has not been shown to be effective in the case of symptomatic obstruction due to strictures [39,40].

Bowel obstruction was observed in patients with CD during the first years following anti-TNF therapy, including patients with inflammatory and fibrotic stenosis. Obstruction may occur in patients with fibrotic strictures for which anti-TNF did not demonstrate a potential efficacy [41].

The therapy with a combination of immunomodulators and anti-TNF increases mucosal healing and remission rates, but it is unable to stop stricture pathogenesis in predisposed patients [42].

Mainly MRI, and CT as a second choice, are the imaging techniques used for evaluation after medical therapy [12]. They are able to quantify the amount of inflammation in a CD pathological loop that is the target of the medical therapy. In fact, imaging techniques are able to compare the amount of edema in the bowel wall (fluid density in CT and hyperintensity in T2-weighted images with and without fat suppression) and the pattern of mural enhancement (stratified or homogeneous) in images acquired before and after medical treatment. MRI and CT are also able to identify fibrotic changes in the pathological loop after medical therapy, in terms of fat density/signal intensity of the submucosal layer and homogeneous enhancement after contrast medium injection, which would explain the reduced efficacy of some drugs after an initial period of therapeutic success [15,17].

Recurrence of strictures can be avoided with medical therapy and quitting smoking after surgery or endoscopic dilatation [43].

Some studies demonstrated that endoscopic dilatation (ED) is a safe and minimally invasive technique for the treatment of strictures, and it preserves bowel length. ED is usually performed after CT/MRI examinations, in order to evaluate the caliber and the extension of the stricture (also using 3D curved multiplanar reconstructions), the number of strictures (in case of multiple stenosis), and concomitant fistulas, which appear as irregular alterations of the mesentery near the pathological loops. ED requires bowel preparation, and it is performed under unconscious sedation or general anesthesia, and under a fluoroscopic guide. Endoscopic dilatation is performed by positioning a guide wire through the stricture on which a high-pressure through-the-scope (TTS) balloon is located. The balloon is subsequently stretched out using water or a contrast agent in order to obtain dilatation of the stricture. The length and diameter of the chosen balloon depend on the size of the stricture and the endoscopic evaluation, on the basis of prior CT/MRI visualization, and measurements of the stricture, including the distance from the ileo-cecal valve/ileo-colic anastomosis based on 2D or 3D multiplanar reconstructions. To date, there is no uniform approach to dilatation regarding balloon dimensions, time and pressure of balloon inflation, number of dilatations, progressive/not progressive dilatation, site and length of stricture, and possibility of crossing with the endoscope after dilatation [44,45].

After balloon dilatation, the endoscopist can have a direct visualization of the mucosa, permitting a precise evaluation of the therapeutic efficacy and early identification and treatment of mucosal changes such as perforations or bleeding [46]. Technical success is usually achieved when the endoscope passes through the stricture, obtaining a caliber of approximately 15 mm.

Some studies demonstrated that the short stricture is the only predictor of an outcome free of surgery, as the balloon length (generally equal to 55 mm) and angulation of some CD tracts may be the cause of endoscopic dilatation failure [47].

CT, MRI, and sometimes ultrasound, are mandatory for evaluation of the stricture after ED, in terms of stricture length, minimum lumen caliber at dilatation point, and diameter of the small bowel loops upstream of the dilatation. These radiological parameters together with clinical data can predict the response to endoscopic therapy [47]. In fact, the procedure was considered successful if the minimum lumen caliber increased after dilatation while the diameter of the loops’ upstream dilatation decreased.

Repeated dilatations are only performed in case of a symptomatic recurrence due to re-stricture confirmed via both endoscopic and radiological evaluation. The association of medical therapy and endoscopic dilatation has demonstrated to be effective in the treatment of CD strictures. To date, there is no evidence regarding the best medical therapy after ED; some studies demonstrated that a combination of azathioprine and budesonide was effective 1 year after the endoscopic procedure. Infliximab can be also used in these cases, with local effectiveness on the strictures and other systemic effects. Furthermore, the combination of ED and medical therapy could be used as a as a bridge to surgery, if needed [47,48].

Isolated, short, and non-fistulizing strictures (reachable from standard endoscope) are the principal indications for ED; the strictures most frequently preferred for treatment are located at the anastomosis after ileo-cecal resection [49].

Some authors observed high complication rates from ED when balloons with larger calibers are used, and in case of multiple dilatations. To avoid complications, in the presence of proctitis or severe anal disease, dilatation of an anorectal stricture cannot be performed, so medical therapy is usually preferred in those patients [49]. CT and MRI are the best imaging techniques for diagnoses of abdominal complications after ED [50]. In particular, CT is the technique of choice for suspected perforation, as it can be performed in the emergency department and it shows free air bubbles in the peritoneal cavity; furthermore, CT can define the degree of perforation, distinguishing perforations confined to the site of ED from those in which air bubbles and free fluid are spread throughout the abdominal cavity.

Unlike ED, the bowel-stenting technique has not yet established itself due to high rate of stent migration and complications. In this case, CT is the imaging technique of choice for the evaluation of stents, being able to identify the position of the stent compared to that of the stricture and the signs of bowel obstruction due to stent migration [51].

## 9. Discussion and Conclusions

MRI and CT are able to answer the clinician’s questions for the radiologist regarding the evaluation of a patient with a CD stricture.

MRE and CTE help to differentiate between inflammatory and fibrotic stenosis, which is fundamental for a correct treatment choice (medical therapy vs. endoscopic dilatation vs. surgery).

A detailed CT or MRI report should include site of the disease, type of stricture (inflammatory vs. fibrotic type), and length of the pathological bowel loop.

We propose the following structured report:

Previous surgery: no, yes. If yes, indicate type.
Wall thickening: no, yes. If present, report:
  - Location: proximal jejunum, distal jejunum, proximal ileum, distal ileum, last ileal loop;
  - Type: symmetric-asymmetric;
  - Degree: mild (<1 cm), moderate (1–2 cm), marked (>2 cm);
  - Distribution: focal (<5 cm), segmental (6–40 cm), diffuse (<40 cm), indicate length;
  - Type of CE after contrast medium: stratified, homogeneous, non-homogeneous, fatty halo sign.
Presence of stenosis: no, yes. If present, indicate lumen caliber.
Upstream loop dilation: no, yes. If present, indicate caliber of the most dilated loop.
Other findings:
Desmoplastic reaction: no, yes.
Lymph nodes: no, yes. If present, report location, morphological characteristics (short axis, CE, necrosis, etc.).
Endo-abdominal fluid flaps: no, yes.
Fistulae: no, yes. If present, indicate type (entero-enteric, entero-cutaneous, other).
Sinus tract: no, yes.
Abscesses/phlegmons: no, yes.If present, report location and diameters.
Fibro-adipose proliferation: no, yes.
Loco-regional hypervascularity: no, yes.

At the end of the report, indicate the subtype (inflammatory, fibro-stenotic, perforating/fistulizing) and compare it with previous radiological exams.

The decision regarding the type of medical treatment helps gastroenterologists to evaluate the possibility of carrying out a minimally invasive dilatation and surgeons to decide to use a resection or another surgical technique, in order to preserve the small bowel. Furthermore, imaging is mandatory for evaluation of the response to the therapy (medical, endoscopic, or surgical) and for diagnosis of complications like bowel obstruction or perforation.

## Figures and Tables

**Table 1:** MRI protocol to study Crohn’s disease.

Sequence	Trade Name	Imaging Plane
Balanced steady-state free procession (bSSFP)	Balanced FFE/TurboFISP/TrueFISP/FIESTA	Axial and coronal
T2-weighted fat-suppressed	FSE/TSE	Axial
3D cinematic bSSFP		Coronal
3D T1-weighted fat-suppressed post-contrast images at 45 and 75 s	VIBE/LAVA	Coronal
Delayed 3D T1-weighted fat-suppressed post-contrast images at 120 s	VIBE/LAVA	Axial
Diffusion-weighted imaging (DWI)		Axial
